# Heavy Alcohol Drinking Associated Akathisia and Management with Quetiapine XR in Alcohol Dependent Patients

**DOI:** 10.1155/2016/6028971

**Published:** 2016-10-25

**Authors:** Zimple Kurlawala, Vatsalya Vatsalya

**Affiliations:** ^1^University of Louisville School of Medicine, University of Louisville, Louisville, KY, USA; ^2^Robley Rex VA Medical Center, Louisville, KY, USA

## Abstract

Heavy drinking contributes to involuntary body movements such as akathisia. Quetiapine has been shown to alleviate symptoms of akathisia; however, its efficacy in the alcohol dependent population is not well established. Thus, we aimed to identify efficacy of Quetiapine in treating akathisia in very heavy drinking alcohol dependent patients. 108 male and female heavy alcohol consuming study participants received 13 weeks of Quetiapine XR. Drinking history (Timeline Followback, TLFB), depression (Montgomery-Asberg Depression Rating Scale, MADRS), and movement (Barnes Akathisia Scale, BARS) measures were collected at baseline (0 W), week 6 (6 W), and week 12 (12 W). The role of drinking, symptoms of depression, and efficacy of Quetiapine for treating akathisia were assessed. In patients with no symptoms of depression (low MADRS), Quetiapine treatment decreased symptoms of akathisia. Patients with clinically significant depression (high MADRS) reported a significant increase in akathisia measures at 6 W which eventually decreased at 12 W to below baseline levels. The increase in akathisia at 6 W corresponded with a significant increase in the patients' total drinks and heavy drinking pattern. Treatment with Quetiapine progressively lowered the occurrence of akathisia in alcohol dependent patients who do not show symptoms of depression. Quetiapine treatment lowered akathisia over time in heavy drinkers who had clinically significant symptoms of depression.

## 1. Introduction

Alcohol dependence is a major public health concern worldwide. Excessive consumption of alcohol over a prolonged period can have adverse neurocognitive effects [1]. Moderate alcohol consumption can transiently improve movement-associated symptoms such as essential tremors [2], dystonias [3, 4], and tics [5]. However, chronic alcohol consumption can trigger or aggravate hyperkinetic conditions such as akathisia, chorea, and myoclonus [6, 7]. Delirium tremens is a severe form of alcohol withdrawal with symptoms of tremors, restlessness, agitation, and altered mental function, peaking around 72 hours after the last drink [8].

The FDA has approved Quetiapine (Seroquel®) for the treatment of schizophrenia, bipolar disorder, and depression. Most clinical trials conducted so far have shown efficacy of Quetiapine to alleviate symptoms of depression, anxiety, and insomnia [9, 10]. Additionally, Quetiapine improved symptoms of akathisia, dyskinesia, and other preexisting involuntary motor movements when used in clinical trials for treating psychotic syndromes, dementia, and Parkinson's disease [11–13]. When compared to Risperidone and Olanzapine in treatment of Parkinson's disease with or without dementia, Quetiapine treatment was just as effective and additionally did not worsen patients' motor symptoms [14]. Compared to a typical antipsychotic such as Chlorpromazine, Quetiapine treated patients showed lower incidence of Parkinsonian symptoms (32% versus 5%) [15].

Patients with concomitant alcohol dependence and depression share a common underlying molecular neuropathology and therefore have similar clinical phenotypic presentation [16]. Alcohol abuse [17, 18] and clinical depression have been individually associated with movement disorders such as akathisia. Fornazzari and Carlen reported development of akathisia in patients during alcohol withdrawal [19]. A large study by Baynes et al. involving 120 subjects reported an association approaching significance (*p* = 0.007) between depression measures and development of akathisia [20]. However, treatment of akathisia in the heavy drinking population with clinically significant depression remains an understudied area. This gap in our knowledge could limit our ability to treat movement disorders in alcoholics who are likely to have symptoms of depression. Using the global assessment item of the BARS scale, Quetiapine was associated with a statistically significant lowering of akathisia [15]. Thus, we aimed to investigate if Quetiapine reduced akathisia in alcohol dependent patients. We also studied effects of history of drinking and active drinking during the treatment course of Quetiapine XR.

## 2. Patients and Methods

### 2.1. Study Participant Population

This study is one of the investigational arms of a larger protocol (ClinicalTrials.gov: NCT#00498628) that was supported by National Institute on Alcohol Abuse and Alcoholism. This investigation evaluated the efficacy of Quetiapine XR in reducing akathisia in alcohol dependent patients with high and low depression scores. A total of 108 men and women received the treatment (Table 1).

The inclusion criteria used in this study were diagnosis of alcohol dependence (using Diagnostic and Statistical Manual of Mental Disorders, Fourth Edition) and age between 18 and 64 years. Men consumed 10 or more drinks per day while women consumed 8 or more drinks per day for at least 40% of the last 60 days of the 90-day drinking assessment (Timeline Followback, TLFB), and all subjects had 0.00 breath alcohol level at the time of consent. Major exclusions included other psychoactive drug dependence within the last year, positive urine screen for drugs, participation in other pharmacological/behavioral studies within the last three months, lifetime diagnosis of major depression or eating disorder, use of antidepressants (last 30 days) and/or antipsychotics (last 14 days) before randomization, and a score ≥10 on the Clinical Institute Withdrawal Assessment of Alcohol.

### 2.2. Procedures and Assessments

Study participants received Quetiapine XR (Seroquel XR® AstraZeneca, Wilmington, DE) for three months in 50 and 200 mg tablets [21]. Clinical and subjective assessments from baseline (0 W), week six (6 W), and end of week 12 (or during week 13, 12 W) were evaluated. The dose of the medication was titrated in the first two weeks up to a target dose of 400 mg/d, which continued through week 12 followed by one week of tapering dose. All individuals received medical management (MM) that included assessment of medication side effects, participant education, and advice on drinking [22].

### 2.3. Data Collection, Statistical Paradigm, and Analysis

Individual demographics were collected including age (years), sex (male or female), weight (lbs.), and drinking (TLFB) history. Age, weight, and recent drinking were included as covariates as needed. We used the following recent TLFB measures [23]: total drinks (TD90); average drinks per drinking day in last 90 days (AvgDPD90); number of drinking days in last 90 days (NDD90); and heavy drinking days in last 90 days (HDD90). Further, there was a 1-month period during the recruitment phase when similar drinking history measures were also collected for 30 days (TD30, HDD30, NDD30, and AvgDPD30). Six-week and twelve-week assessment of drinking history were collected using TLFB for the 2-week interval prior to the visit.

Patients were categorized by their MADRS scores at baseline (high MADRS or clinically significant if > seven; and low MADRS or clinically nonsignificant level if ≤ seven) [24]. The presence of clinically significant MADRS scores was used as the primary independent variable. We compared the prevalence of akathisia as the primary study outcome in patients with clinically significant and clinically nonsignificant depression and used the Barnes Akathisia Rating Scale (BARS) instrument for collecting data on akathisia. BARS was designed to rate the severity of drug-induced or Parkinson's disease-based akathisia and has been used regularly since its adoption in 1989 in clinical studies involving assessment of akathisia. Quantitative assessment of akathisia is possible when both the subjective experience and the objective signs and features are taken into account [25]. To test the effect of time, we evaluated prevalence of akathisia during the treatment period using Chi-square analysis. We used univariate analysis of variance (UANOVA) to characterize recent drinking at each time point between the two depression groups (used as a factor) and further with the prevalence of reported akathisia within each MADRS group. We also report corresponding Clinical Institute Withdrawal Assessment of Alcohol (CIWA) scores for these patients. We also performed repeated analysis of variance between baseline, week 6, and end of the study assessment of akathisia between low MADRS and high MADRS groups. Data analysis platforms used in this study were SPSS 22.0 version (IBM, Chicago, IL), MS Office Excel 2013 (MS Corp., Redmond, WA), and GraphPad prism 6 (GraphPad Software, Inc., La Jolla, CA). The level of statistical significance was set at *p* ≤ 0.05. Statistical significance or trend levels are noted as needed.

### 2.4. Study Limitations

There were three times as many men as women in this study, but since this occurred in all groups evenly, it did not restrain the analysis; however between sex analysis could not be performed. There were a few dropouts during the course of the study and 4-5% of the total data points could not be included from this study due to missing values. Statistical analysis data were not conclusive at week 12 due to lowered prevalence of akathisia since by then their symptoms most likely got better with treatment.

## 3. Results and Discussion

### 3.1. Characterization of Patients and Drinking History

Out of 108 enrolled patients, 31 patients showed clinically significant level of depression using the MADRS scale. Demographic measures were comparatively similar in all subgroups differentiated by MADRS group (Table 1). Patients consuming at least 10 drinks per day matched the profile of very heavy drinkers [26]. There were more males and they weighed more than females in both MADRS groups. There was no significant main effect of any of the demographic or drinking history markers between the two MADRS groups, and these patients also roughly matched in age and drinking history. We found that Quetiapine reduced the occurrence of akathisia in very heavy drinking patient population in the group having clinically significant depression (high MADRS scores). CIWA scores for the assessment of alcohol withdrawal severity did not show any clinical significance throughout the study period (Table 1). At baseline, there was a statistically significant elevation of CIWA scores found in patients with high MADRS; however numerically this elevation was not clinically significant.

### 3.2. Prevalence and Characterization of the Akathisia Measures

We performed repeated analysis of variance to measure severity of akathisia using BARS assessment at week 6 and week 12 comparing from baseline values to identify if there is any significant contrast existing between the timelines due to MADRS reporting. We found that there was a significant contrast of time, *p* ≤ 0.001. This finding supports our assessment that time course of treatment has a significant effect on improvement of akathisia, demonstrated by gradual lowering of extent of reporting of akathisia in both MADRS groups. Notably, there was also a trending level of contrast effect in time and MADRS severity for akathisia reporting, *p* = 0.105. This suggested that Quetiapine was actively reducing akathisia symptoms in both groups. However, there was a trending difference between the two groups, for which assessment of prevalence of akathisia at each time point was tested further. We performed Chi-square analysis to separate this effect of Quetiapine treatment on occurrence of akathisia between the two groups. Also, there was no change in the significance observed when drinking history markers were included. Other studies that have studied the efficacy of Propranolol [27] and Lorazepam [28] for treatment of akathisia have also reported time-course dependent alleviation of akathisia symptoms. There is substantial evidence that 5-HT2A antagonists are successful in treating akathisia symptoms, exclusively neuroleptic-induced akathisia [29]. A part of the subject population in this study reported symptoms of akathisia from alcohol withdrawal regardless of baseline level of MADRS. Our data suggest that continued treatment with Quetiapine XR improved symptoms of akathisia especially in patients with clinically significant depression (high MADRS), when there was simultaneous lowering of alcohol intake.

**Figure 1:**
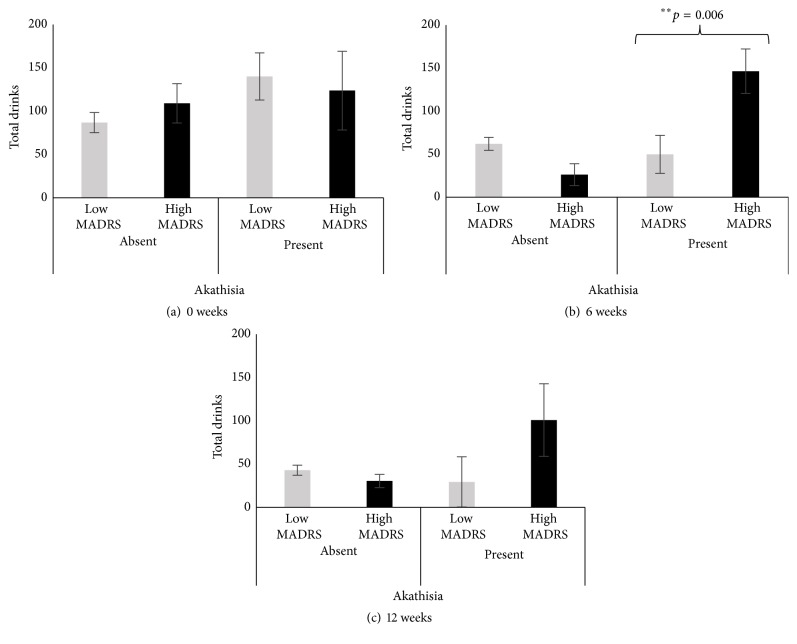
Level of drinking in alcohol dependent patients by MADRS group and reporting of akathisia. (a) Baseline total drinks (TD30) by MADRS and akathisia. (b) Total drinks (TD weeks 4–6) at week 6 by MADRS and akathisia. (c) Total drinks (TD weeks 10–12) at week 12 by MADRS and akathisia. Data presented as M ± SE (mean with standard error). Significance was set at *p* ≤ 0.05.

Thereafter, we conducted Chi-square tests to evaluate significance of occurrence of akathisia during Quetiapine treatment course by depression level (Table 2). At baseline, patients in both MADRS groups experienced akathisia symptoms; however, there were no significant differences between the two groups. The occurrence of akathisia more than doubled at 6 W from baseline reporting (0 W) in the high MADRS group. Interestingly, akathisia incidence (44.4%) in high MADRS group at week 6 was also four times higher than the corresponding occurrence of akathisia in low MADRS group (Table 2). Our data are consistent with a recently published study, investigating efficacy of Quetiapine in treating alcohol dependence in patients diagnosed with bipolar disorder. The authors found that although Quetiapine treatment did not yield anticipated lowering of alcohol intake, patients experienced an increase in akathisia symptoms at 6 weeks (BARS score, *p* = 0.04), but not at 12 weeks [30]. At baseline, the likelihood of akathisia incidence in high MADRS group compared to low MADRS group was low. However, at week 6, this likelihood increased to significant moderate levels of occurrence in the same comparison that was also statistically significant (Table 2). At baseline and at 12 W, the likelihood ratio between the two groups remained low, tested by Chi-square analyses. At 12 weeks, the incidence of akathisia in high MADRS group dropped to 9%, a ~5-fold decrease from 6 W and 2-fold decrease from baseline. Patients, who exhibited lowering of akathisia scores after 6 weeks of treatment, also had correspondingly lowered MADRS scores. This finding could be explained by the lowered levels of the heavy drinking markers (Figure 1(c)).

In the low MADRS group, the occurrence of akathisia continuously dropped during the entire treatment course with a 5-fold lowering at 12 W from the baseline occurrence (0 W), which also supports the role of Quetiapine in reducing akathisia in heavy drinking alcoholic patients without any ongoing clinically significant depression, who were actively drinking. Thus, the primary aim of this study was to determine if Quetiapine XR reduces akathisia in alcohol dependent patients. We found that it did in both depression groups. Akathisia is generally associated with the sense of inner restlessness, mental uneasiness, unrest, or dysphonia, which can be intense [31]. The occurrence and reduction in akathisia have also been evaluated in several studies that have used atypical antipsychotics including Clozapine [22], Olanzapine [23], Quetiapine [24–26], Ziprasidone [27], and others.

Based on our findings, we also report that there was an increase in akathisia in high MADRS group at 6 weeks of treatment, and it was important to identify the cause for such an increase. Akathisia has also been reported to be either primary or secondary to drug use [28, 29]. It is known that alterations in movement can be provoked by alcohol intake [32]. We found an increase in akathisia reported at 6 weeks and evaluated recent drinking amount and drinking pattern at baseline, 6 W, and 12 W time points to identify whether this increase was due to a direct adverse effect of Quetiapine treatment, a change in drinking pattern, or interaction of Quetiapine with drinking in the high MADRS group.

Total drinks (marker of heavy drinking) recorded at all time points were higher in patients who exhibited akathisia in the high MADRS group. We did not find any significant differences in drinking either at baseline (0 W) or at the end of the study (12 W) between patients that exhibited akathisia and those that did not (Figures 1(a) and 1(c)). However, at 6 weeks of treatment, total drinks reported were approximately four times more in patients who exhibited akathisia symptoms in the high MADRS group (Figure 1(b)) and were highly statistically significant. The amount of drinking potentially influenced the incidence of akathisia at 6 W in high MADRS group. A sizeable proportion of patients with depression and related disease conditions reported higher susceptibility to heavy alcohol drinking [33]. Interestingly, in the patient group with low MADRS scores, total drinks decreased over time, and patients who exhibited akathisia did not show significant differences in total drinks compared to those who did not have akathisia, asserting the role of drinking and clinically relevant level of MADRS in the appearance of akathisia symptoms at 6 weeks in the high MADRS group.

**Figure 2:**
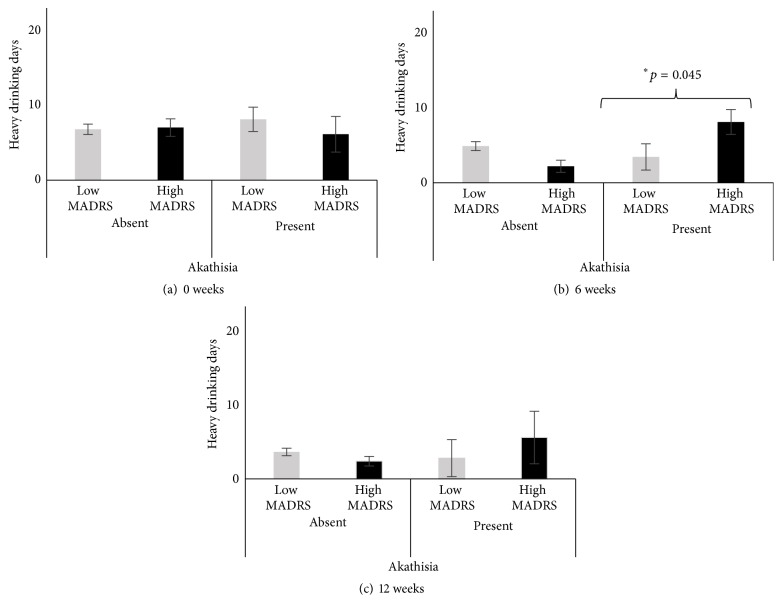
Frequency of heavy drinking days (HDD) in alcohol dependent patients by MADRS and reporting of akathisia. (a) Baseline (0 weeks) frequency of heavy drinking days (HDD30) by MADRS and akathisia. (b) Heavy drinking days (TD weeks 4–6) at week 6 by MADRS and akathisia. (c) Heavy drinking days (TD weeks 10–12) at week 12 by MADRS and akathisia. Data presented as M ± SE (mean with standard error). Significance was set at *p* ≤ 0.05.

Heavy drinking days (another marker of heavy drinking from TLFB questionnaire, HDD30) at baseline were higher in patients with akathisia in the high MADRS group. However, they were higher at both 6-week and 12-week assessments (Figures 2(a) and 2(c)). HDD at 6 weeks showed a significant elevation in patients who exhibited akathisia (Figure 2(b)) compared to those who did not, within the high MADRS group. Within the low MADRS group, total number of heavy drinking days progressively lowered as treatment continued and frequency of heavy drinking days was similar in patients with akathisia compared to those without akathisia.

Both the amount and pattern of heavy drinking were influential in increasing the incidence of akathisia in the high MADRS group at six weeks. The amount and pattern of heavy drinking were lower in this group both at baseline and at 12 weeks of treatment. On the other hand, in the low MADRS group, we did not find any significant increase in patterns of heavy drinking during treatment, and the occurrence of akathisia progressively decreased over time. Thus, we did not observe any influence of drinking alone or its interaction with Quetiapine in patients who did not have clinically significant symptoms of depression. No other markers of drinking showed any significant differences between the two groups. We did not find any interaction between MADRS groups and the presence of akathisia.

## 4. Conclusions

Quetiapine XR seemed to alleviate akathisia symptoms in very heavy drinking alcohol dependent (AD) patients who did not have clinically significant symptoms of depression. In alcohol dependent patients with clinically significant symptoms of depression, the effects of Quetiapine XR in reducing akathisia do not seem to begin until after the first six weeks of treatment. Increases in the amount and pattern of heavy drinking potentially played a significant role in exacerbation of akathisia symptoms in heavy drinking AD patients. However, with continued treatment of Quetiapine XR, AD patients showed a significant lowering of the incidence of akathisia. Results from the present study extend our understanding of the interplay between heavy drinking, presence of clinically significant symptoms of depression, and the efficacy of Quetiapine XR in alleviating symptoms of akathisia in very heavy drinking alcohol dependent patients. Akathisia has been reported to either worsen or emerge as a new symptom during alcohol withdrawal in chronic alcoholics [19]. Alcohol dependence is known to be a frequent comorbid condition in depressed patients [34]. Results from our study show that an antidepressant drug such as Quetiapine XR may reduce akathisia potentially resulting from alcohol withdrawal.

## Figures and Tables

**Table 1:** Baseline demographics, drinking history markers, and acute withdrawal assessment in alcohol dependent patients by MADRS group and gender.

Treatment and measures	Low MADRS (clinically nonsignificant)			High MADRS (clinically significant)			Significance between the MADRS grs. (*p* < 0.05)
	Males (63)	Females (14)	Overall (77)	Males (27)	Females (4)	Overall (31)	
Age (yrs.)	44.4 ± 10.0	49.9 ± 8.5	45.43 ± 9.919	46.0 ± 8.1	41.8 ± 4.3	45.42 ± 7.749	NS
Weight (lb.)	194.9 ± 40.8	153.2 ± 27.6	187.34 ± 41.8	195.1 ± 35.0	174.3 ± 35.4	192.4 ± 35.2	NS

*Drinking history*							
TD90	1268.9 ± 503.5	1030.0 ± 507.8	1225.5 ± 509.4	1370.0 ± 587.3	1064.8 ± 309.1	1328.8 ± 564.9	NS
AvgDPD90	14.1 ± 5.6	11.4 ± 5.6	13.6 ± 5.6	15.2 ± 6.5	11.8 ± 3.4	14.8 ± 6.2	NS
HDD90	63.1 ± 22.7	72.1 ± 13.1	64.8 ± 21.5	67.7 ± 24.7	76.0 ± 16.2	68.7 ± 23.7	NS
NDD90	80.5 ± 15.1	81.3 ± 13.7	80.62 ± 14.7	79.1 ± 13.6	83.8 ± 6.5	79.7 ± 12.9	NS

*Acute withdrawal assessment*							
CIWA BL	1.7 ± 2.0	1.1 ± 2.4	1.6 ± 2.1	3.6 ± 2.6	3.5 ± 3.5	3.6 ± 2.7	≤0.001
CIWA 6 W	0.9 ± 1.3	0.6 ± 1.2	0.9 ± 1.2	1.1 ± 1.4	0.7 ± 1.2	1.0 ± 1.3	NS
CIWA 12 W	1.1 ± 1.4	0.1 ± 0.3	0.9 ± 1.4	1.1 ± 1.6	1.7 ± 0.6	1.2 ± 1.5	NS

TD90: total drinks in 90 days; AvgDPD90: average drinks per drinking day in last 90 days; HDD90: heavy drinking days in last 90 days; NDD90: number of drinking days in last 90 days.

**Table 2:** Comparison of reported akathisia in alcohol dependent patients with clinically significant (high) and clinically nonsignificant (low) MADRS at baseline (0 weeks) and 6-week and 12-week assessment timelines.

Week/s	% akathisia incidence		Likelihood ratio	*p *value	Probability
	Low MADRS	High MADRS			
0	15.6	19.7	0.221	0.638	Low
6	11.9	44.4	5.108	0.024	Moderate
12	3.7	9.1	0.583	0.445	Low

## Abbreviations

Quetiapine XR: Quetiapine fumarate XR
AD: Alcohol dependents
MADRS: Montgomery-Asberg Depression Rating Scale
BARS: Barnes Akathisia Rating Scale
TLFB: Timeline Followback
MM: Medical management
Baseline: 0 W
Steady state: 6 W
Study endpoint: 12 W.
